# MEK-inhibitor PD184352 enhances the radiosensitizing effect of the Hsp90 inhibitor NVP-AUY922: the role of cell type and drug-irradiation schedule

**DOI:** 10.18632/oncotarget.26436

**Published:** 2018-12-21

**Authors:** Felix Grabenbauer, Astrid Katzer, Dmitri Sisario, Simon Memmel, Michael Flentje, Vladimir L. Sukhorukov, Cholpon S. Djuzenova

**Affiliations:** ^1^ Department of Radiation Oncology, University Hospital of Würzburg, Würzburg, Germany; ^2^ Department of Biotechnology and Biophysics, University of Würzburg, Würzburg, Germany

**Keywords:** cell cycle arrest, colony survival, DNA damage, histone γH2AX, radiation sensitivity

## Abstract

Targeting MEK protein in cancer cells usually leads to acquired resistance to MEK inhibitors and activation of the prosurvival protein Akt. Since both MEK and Akt are clients of the Hsp90 chaperone system, the present study explores the responses of irradiated lung carcinoma A549 and glioblastoma SNB19 cell lines to combined MEK and Hsp90 inhibition. Unexpectedly, the MEK inhibitor PD184352 administered 24 h prior to irradiation, enhanced cell survival through upregulation of not only MEK and Erk1/2 but also of Akt. In contrast, PD184352 added 1 h before irradiation strongly reduced the expression of Erk and did not upregulate Akt in both cell lines. As a result, the MEK inhibitor increased the radiosensitizing effect of the Hsp90 inhibitor NVP-AUY922 in glioblastoma SNB19 cells. Possible reasons for the enhanced cell killing under this short-term pretreatment schedule may be a down-regulation of Erk during or directly after irradiation, increased DNA damage and/or a strong G_2_/M arrest 24 h after irradiation. In addition, an 1-h pretreatment with PD184352 and/or NVP-AUY922 under schedule II induced neither G_1_ arrest nor up-regulation of p-Akt in both cell lines as it did under schedule I. Yet, a long-term treatment with the MEK inhibitor alone caused a strong cytostatical effect. We conclude that the duration of drug pretreatment before irradiation plays a key role in the targeting of MEK in tumor cells. However, due to an aberrant activation of prosurvival proteins, the therapeutic window needs to be carefully defined, or a combination of inhibitors should be considered.

## INTRODUCTION

More than 90% of tumors harbor an oncogenic mutant *kRAS* (rat sarcoma protein), whose aberrant activation results in the activation of the RAF (rat fibrosarcoma) protein family of serine/threonine kinases, which, in turn, activate the mitogen-activated protein kinase (MAPK) kinase (MEK) and the extracellular signal-regulated kinase (Erk). As a result, activated Erk phosphorylates its target substrates thus promoting tumor cell proliferation, survival and migration, along with conferring resistance to radio- and chemotherapy [1, 2]. Therefore, new therapeutic approaches and agents are currently needed to sensitize malignant cells to radiation and/or chemotherapy.

Lying downstream of RAS and RAF and directly upstream of Erk, the protein kinase MEK occupies a critical signaling node, and its inhibitors have been the subject of intense drug discovery efforts [3]. A number of MEK inhibitors have shown promising outcome in preclinical studies and clinical trials [4–6]. In particular, the novel ATP non-competitive MEK inhibitor AZD6244 has demonstrated high specificity and anti-proliferative activity in *in vitro* and *in vivo* models [7]. Several studies have shown that in addition to the cytostatic effects AZD6244 also sensitizes human tumor cell lines of different origins to ionizing radiation (IR), underlining the potential of the MAPK pathway as a target for radiosensitization [4, 8, 9]. However, one of the major drawbacks of the inhibition of MEK alone is the induction of a feedback loop leading to elevated levels of MEK protein [10]. Furthermore, because of the mutual dependence of MAPK- and PI3K-pathways, MEK inhibition causes a concomitant up-regulation of p-Akt [11], which is also known to increase survival, growth, radio- and chemoresistance of cells [12], thus counteracting tumor therapy.

Interestingly, both MEK and Akt proteins are clients of the heat shock protein 90 (Hsp90) chaperone system, which consists of ubiquitously and abundantly expressed polypeptides required for the energy-driven stabilization, conformation and function of a large number of cellular proteins, termed Hsp90 clients [13]. Among many functions, Hsp90 clients contribute to the pathways involved in the induction of MAPK and nuclear factor-kappa B (NF-κB) [14, 15]. Hsp90 also stabilizes Raf-1, Akt, and ErbB2 proteins, which are associated with protection against radiation-induced cell death [16, 17].

Considering the above mentioned functions of Hsp90, its inhibition can be a promising strategy for implementing a multi-targeted approach to radiosensitization of cancer cells. A number of studies including our own [18–20] have already explored Hsp90 as a potential molecular target for radiosensitization of tumor cell lines derived from a variety of histologies, including glioma, prostate and lung carcinoma.

In order to prevent the adverse up-regulation of p-MEK and p-Akt we make use in the present study of the fact that both proteins are clients of the Hsp90 chaperone system [13]. Therefore, in addition to the MEK inhibitor PD184352 we also used a very efficient inhibitor of Hsp90, NVP-AUY922, which is known to significantly enhance the radiosensitivity of various tumor cell lines [19]. We first examined whether the MEK-inhibitor-mediated up-regulation of p-MEK and p-Akt can be prevented by the Hsp90 inhibitor. Secondly, we tested whether MEK inhibition can enhance the radiosensitizing effect of the Hsp90 inhibitor in the lung carcinoma A549 and glioblastoma SNB19 cell lines. To inhibit MEK we used an ATP non-competitive MEK1/2 inhibitor PD184352, an anti-tumor drug with low toxicity which was the first MEK1/2 inhibitor to enter into a clinical trial [21].

## RESULTS

The following experiments were designed to evaluate the effects of PD184352 and NVP-AUY922 on the radiation sensitivity, marker protein expression, DNA damage/repair and cell cycle progression of 2 tumor cell lines. Each compound was applied either alone or in combination. Two drug-IR treatment protocols differing in the timing of irradiation relative to drug application were examined (Supplementary Figure 1). In the long-term pretreatment protocol (hereafter referred to as Schedule I), the substances were added 24 h before IR and washed out shortly before IR. In the short-term pretreatment protocol (Schedule II), the drugs were added 1 h prior to IR and remained in CGM up to 24 h post-IR.

### Effects of PD184352 and NVP-AUY922 on colony survival after IR

**Figure 1:**
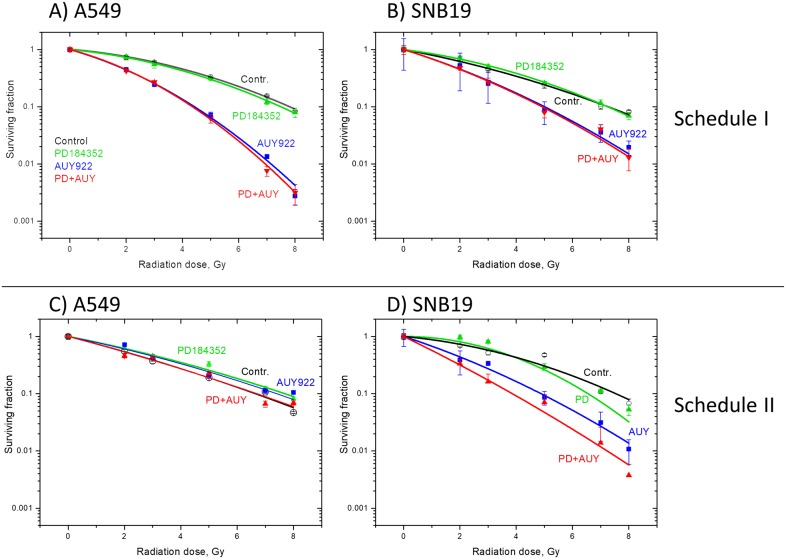
Clonogenic survival of A549 **(A, C)** and SNB19 **(B, D)** tumor cell lines treated with PD184352 and NVP-AUY922 for either 24 h (A, B) or 1 h (C, D) before IR. Irradiated cells were plated for the colony-forming test either immediately (A, B) or 24 h (C, D) after IR. After 10-12 days, colonies containing at least 50 cells were scored as survivors. Data derived from at least three independent experiments for each cell line were pooled together and fitted by a linear quadratic equation (Equation 1). The SD values are indicated by error bars.

Figure 1 shows the cell survival curves of drug-treated cells plotted *versus* the radiation dose, along with the best fits of the linear-quadratic (LQ) model (Equation 1) to the data. The plating efficiencies (PE) of non-irradiated cell samples, as well as the fitted parameters derived with the LQ model, including the surviving fraction at 2 Gy (SF2), the radiation dose required to reduce colony forming ability by 90% (D_10_) and the growth inhibition factor (IF_10_) are summarized from 5 independent experiments in the Supplementary Tables 1 and 2. As seen in Figure 1A, 1B, a 24-h-pretreatment with PD184352 alone did not radiosensitize the tested tumor cells at all (green *vs*. black curves). In contrast, NVP-AUY922 strongly radiosensitized both cell lines under Schedule I (Figure 1A, 1B, blue curves). Interestingly, concomitant addition of PD184352 under Schedule I did not affect the radiosensitizing effect of NVP-AUY922 in both cell lines (Figure 1A, 1B, red curves).

Next we incubated tumor cells with both drugs for a short duration (1 h) before IR (Schedule II, Supplementary Figure 1) and for 24 h post-IR before seeding for the colony test. As with the prolonged incubation before IR (Schedule I), the radiation sensitivities of both cell lines pretreated with PD184352 alone according to Schedule II remained unchanged as compared to controls (green *vs*. black curves in Figure 1C, 1D). We also found that under Schedule II the Hsp90 inhibitor did not radiosensitize A549 cells (Figure 1C, blue curve), which is in agreement with our previously published data [20]. However, under Schedule II concomitant presence of PD184352 moderately increased the radiosensitizing effect of NVP-AUY922 in SNB19 cells (Figure 1D, red *vs*. blue curves). The effect is also evident from the moderately reduced SF2 and D_10_, and increased IF_10_ values in irradiated SNB19 cells treated with both drugs (Supplementary Table 2).

### Effects of inhibitors and irradiation on the expression of marker proteins

**Figure 2:**
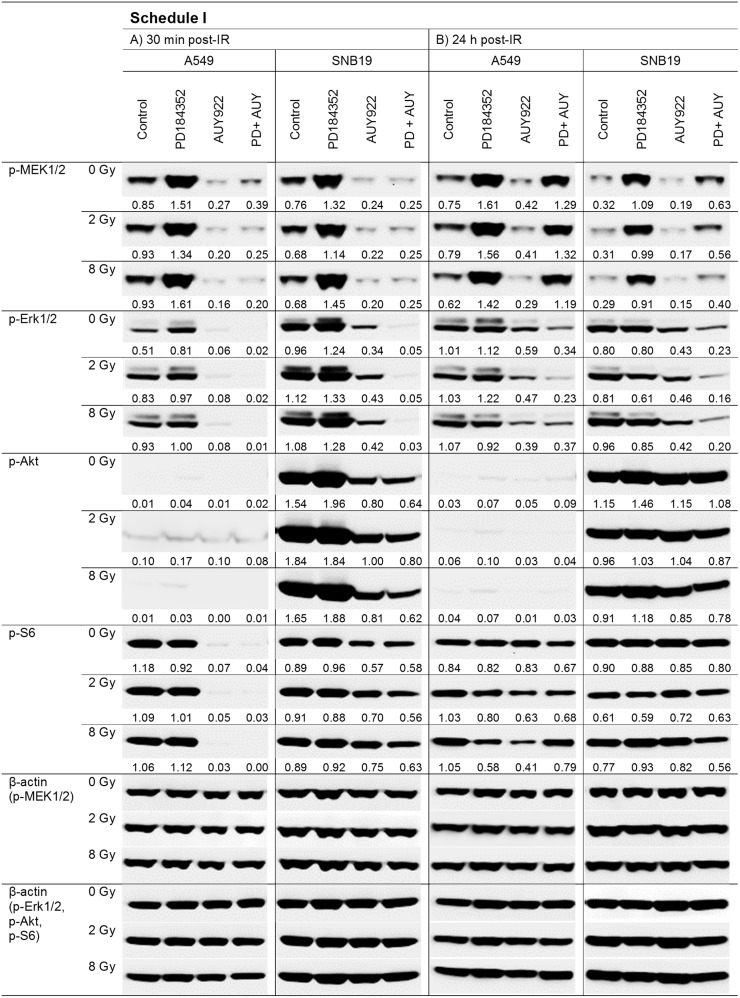
Representative Western blot analysis of expression levels of several marker proteins in A549 and SNB19 tumor cells detected either 30 min (LHS, left-hand side) or 24 h (RHS, right-hand side) post-IR with 2 and 8 Gy Cells were treated with inhibitors 24 h before IR (Schedule I). Each protein band was normalized to the intensity of β-actin used as loading control, and the ratios are denoted numerically if significant changes in the expression are present. The experiment was repeated at least three times.

**Figure 3:**
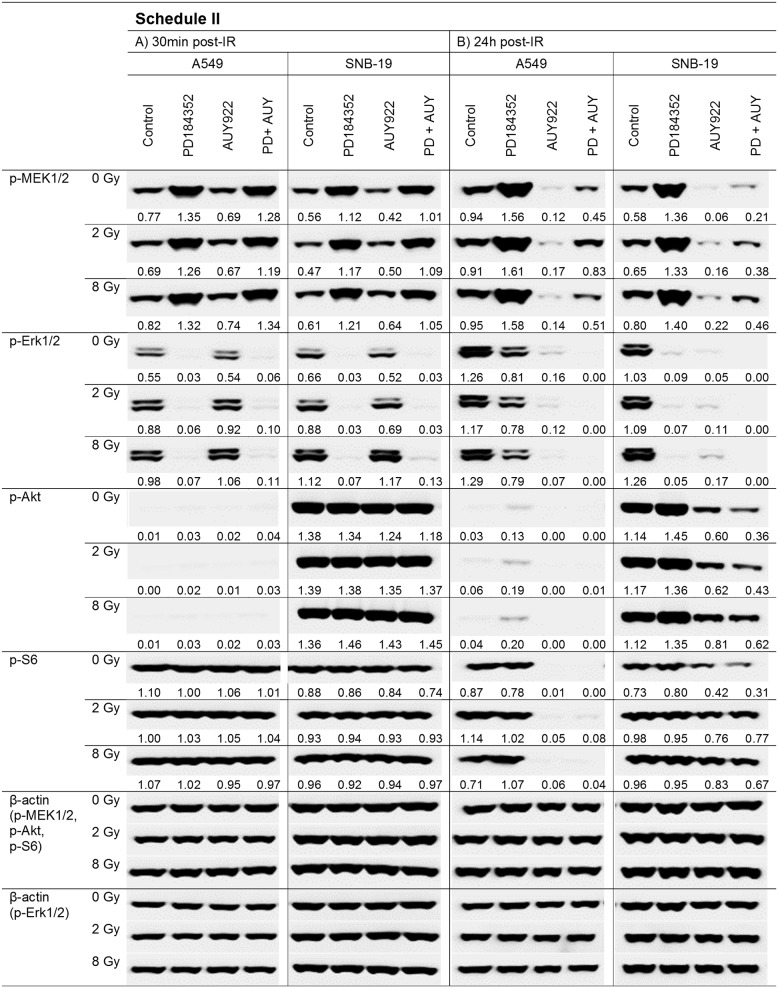
Representative Western blot analysis of expression levels of several marker proteins in A549 and SNB19 tumor cells detected either 30 min (LHS) or 24 h (RHS) post-IR with 2 and 8 Gy Cells were treated with inhibitors 1 h before IR (Schedule II) and incubated with the substances 24 h post-IR. Each protein band was normalized to the intensity of β-actin used as loading control, and the ratios are denoted numerically if significant changes in the expression are present. The experiment was repeated at least three times.

To elucidate the molecular basis for the distinct radiation responses of tumor cells subjected to different drug-IR treatment schedules (Figure 1), we analyzed by western blotting the expression of several marker proteins after treatment with the MEK inhibitor alone or in combination with the Hsp90 inhibitor. The MEK inhibitor PD184352 can be expected to suppress the MAPK pathway, which is frequently mutated in tumor cells [22] thus promoting cell survival, proliferation and migration [23]. Figures 2 and 3 show exemplarily the western blot data of control and drug-treated samples of both cell lines probed for the marker proteins detected in cell samples treated according to Schedule I and II, respectively. Samples of both cell lines shown on the left- and right-hand sides (LHS, RHS) of Figures 2 and 3 were prepared 30 min and 24 h post-IR (2 and 8 Gy), respectively.

As seen in Figure 2, long-term incubation with PD184352 (Schedule I) strongly upregulates the expression of p-MEK1/2 in both cell lines. The high levels of p-MEK1/2 persisted even after washing out the substance and this effect was independent of IR. Likewise, PD184352 strongly up-regulated p-Erk1/2 shortly after IR, although to a lesser extent than p-MEK1/2. In contrast to p-MEK1/2, the expression of p-Erk1/2 nearly returned to control levels 24 h post-IR.

It is obvious from Figure 2 (30 min post-IR) that both cell lines treated with the Hsp90 inhibitor NVP-AUY922 either alone or in combination under Schedule I were irreversibly depleted of p-MEK1/2, a client of Hsp90. The expression of another Hsp90 client, p-Erk1/2, was also completely abolished in A549 cells treated with NVP-AUY922 alone or in combination. In SNB19 cells the expression of p-Erk1/2 was either reduced or completely abolished after Hsp90 inhibition alone or in combination, respectively.

Because of the known crosstalk between the MAPK and PI3K pathways [24], we also analyzed two marker proteins of the PI3K pathway, *i.e.* p-Akt and p-S6. In fact, we found that long-term incubation with the MEK inhibitor alone slightly increased the expression of p-Akt (Figure 2) in non-irradiated as well as in irradiated SNB19 cells. However, after washing out the inhibitor the effect was less evident. Addition of the Hsp90 inhibitor alone or in combination reduced the expression of p-Akt, which is also a client of Hsp90. Yet its reduction was not as strong as the observed depletion of the two other Hsp90 clients, p-MEK1/2 and p-Erk1/2. Interestingly, the two tested cells differed markedly in the background expression of p-Akt, *i.e*. barely detectable in A549 cells *vs*. highly expressed in SNB19 cells. This apparent compensatory activation of the PI3K pathway can be explained by the lack of *PTEN* in SNB19 cells.

To further elucidate possible changes in the PI3K pathway in drug treated cells we analyzed the expression of the proliferation marker p-S6 [25]. We found that the MEK inhibitor did not affect p-S6 expression in both cell lines. In contrast, the Hsp90 inhibitor NVP-AUY922 completely depleted A549 cells of p-S6 and moderately reduced the expression of this protein in SNB19 cells (Figure 2).

We also analyzed the expression of the above mentioned proteins in cells subjected to drug-IR treatment according to Schedule II, *i.e*. with inhibitors added 1 h before IR (Figure 3). In this case, the expression of p-MEK1/2 was strongly increased in both cell lines after addition of PD184352 alone or in combination with NVP-AUY922 (Figure 3, LHS). Interestingly, the same samples of both cell lines were completely depleted of p-Erk1/2. As expected, short-term pretreatment with the Hsp90 inhibitor did not change the expression of both proteins detected 30 min post-IR. Twenty four hours after IR (Figure 3, RHS), the expression of p-MEK1/2 was still increased in both cell lines treated with the MEK inhibitor alone. At the same time SNB19 cells were still depleted of p-Erk1/2, whereas in A549 cells p-Erk1/2 recovered to 60-70% of the control level. Prolonged treatment with NVP-AUY922 alone or in combination caused depletion of p-Erk1/2 in both cell lines (Figure 3, RHS, 24 h post-IR). The depletion of p-MEK1/2 was less evident, especially after combined drug treatment.

As seen in Figure 3 (LHS, 30 min post-IR), the levels of p-Akt and p-S6 remained unchanged 30 min post-IR in all cell samples treated according to Schedule II. This suggests that the adverse up-regulation of p-Akt induced by prolonged MEK inhibition according to Schedule I (Figure 2) can be prevented by shortening the time of drug application prior to IR. As expected, extending post-IR drug application to 24 h (Figure 3, RHS) gave rise to a protein expression pattern similar to that observed in cells treated according to Schedule I (Figure 2, LHS). The expression of tested proteins was not affected by IR.

In addition, we detected the expression of non-phosphorylated forms of MEK1/2, Erk1/2, Akt and S6 proteins. As seen in Supplementary Figures 2, 3, contrary to the phosphorylated forms, the expression of non-phosphorylated forms of MEK1/2, Erk1/2, Akt and S6 was only moderately repressed after addition of the Hsp90 inhibitor alone or in combination. At the same time, the expression of proteins was not changed at all after addition of MEK inhibitor.

Representative Western blots of Hsp90 and Hsp70 expression in both tumor cell lines treated with PD184352 or NVP-AUY922, or both substances are shown in Supplementary Figure 4 (Schedule I) and Supplementary Figure 5 (Schedule II). As evident from the figures, PD184352 alone exerted little (if any) effect on the expression levels of Hsp90 and Hsp70, as compared to untreated control. In contrast, treatment with the Hsp90 inhibitor NVP-AUY922 considerably increased the levels of Hsp70 (and to lesser extents of Hsp90) in both tested cell lines.

### Impact of PD184352 and NVP-AUY922 on IR-induced DNA damage

**Figure 4:**
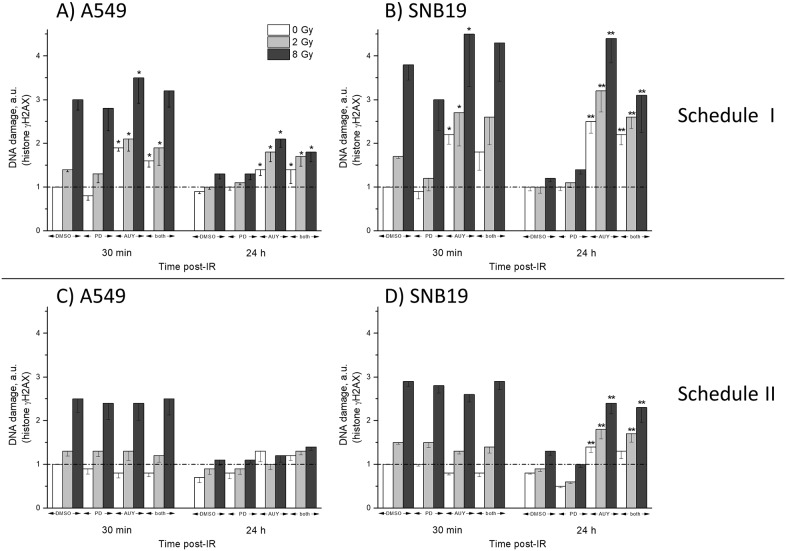
DNA damage in A549 **(A, C)** and SNB19 **(B, D)** cells assessed by histone γH2AX and quantified by flow cytometry 30 min and 24 h post-IR. Top and bottom halves of the graph refers to the Schedule I and II, respectively. The bar graphs represent the means (± SD) of at least 3 independent experiments. The data of each cell line are normalized to the initial γH2AX content (at 0.5 h post-IR) detected in drug-free non-irradiated controls.

To elucidate the reasons for the different radiation responses of cells subjected to drug-IR treatments according to schedules I and II in colony-forming test (Figure 1), we further evaluated IR-induced DNA damage in control and drug-treated cells. The induction of DNA double-strand breaks (DSBs) was analyzed by the expression of phosphorylated histone H2AX, *i.e*. γH2AX, [26] (Figure 4) after irradiation of tumor cells, either non-treated or pretreated with inhibitors.

Figure 4 shows the values of DNA damage detected by γH2AX expression in 4 independent experiments, conducted following both drug-IR schedules, 30 min and 24 h post-IR. As expected, the radiation-induced DNA damage increased with radiation dose. Interestingly, in cells pretreated 24 h with PD184352 according to Schedule I the damage was similar to or even lower than in the respective DMSO-treated controls (Figure 4A, 4B). Under Schedule I, the highest DNA damage was observed 30 min post-IR in samples treated with NVP-AUY922 and IR, most notably in SNB19 cells (Figure 4B). Moreover, the Hsp90 inhibitor administered alone under Schedule I strongly affected the DNA damage repair process in both tumor cell lines (Figure 4A, 4B), which is reflected by the much slower clearance of γH2AX 24 h after IR, as compared to that of drug-free irradiated samples. The combination of both substances and IR caused somewhat lower DNA damage, as compared to NVP-AUY922 alone. The residual DNA damage in irradiated samples of both cell lines treated with the combination of the two inhibitors was lower than in cells treated with the Hsp90 inhibitor alone, but it was still much higher than in the drug-free irradiated samples.

On the other side, if a combination of both substances was added shortly (1 h) before IR and kept for 24 h thereafter (Schedule II, Figure 4C, 4D), both induced and residual DNA damage in irradiated and drug-treated A549 cells were almost identical to the irradiated drug-free samples (Figure 4C). In contrast, despite similar initial DNA damage in drug-free and drug-treated SNB19 cells (Figure 4D, 30 min, 8 Gy), the DNA damage repair occurred much more slowly in samples treated with NVP-AUY922 alone (or in combination with PD184352) than in drug-free controls (Figure 4D, 24 h, 8 Gy).

### Effects of inhibitors and IR on cell-cycle progression

**Figure 5:**
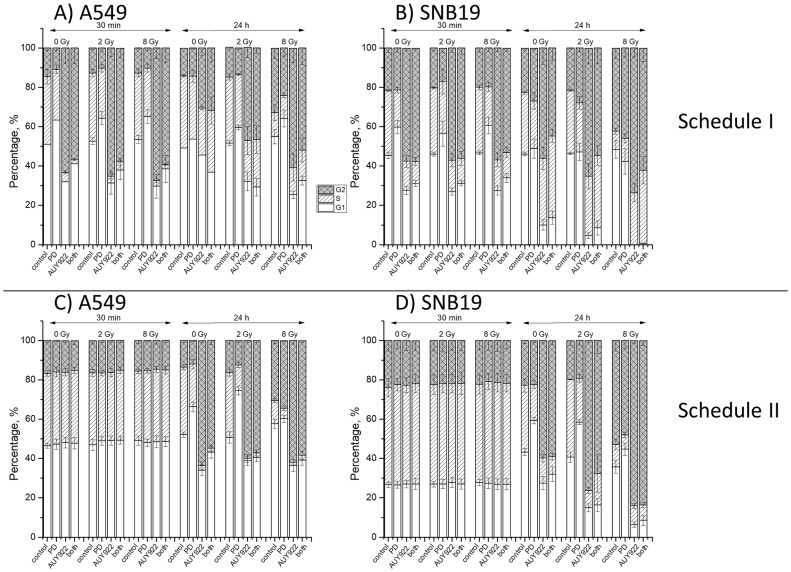
Cell cycle-phase distribution in A549 **(A, C)** and SNB19 **(B, D)** tumor cells treated with PD184352, NVP-AUY922 either alone or in combination and irradiated with 2 and 8 Gy. Top and bottom halves of the graph refers to Schedule I and II, respectively. Thirty minutes and 24 h after IR cells were fixed, permeabilized, stained with propidium iodide, and analyzed for DNA content by flow cytometry. Data are presented as means (± SE) of at least three independent experiments. For details see Supplementary Tables 3-6.

By assessing the possible impact of both drugs and IR on the cell-cycle progression, we further attempted to dissect the mechanisms underlying the moderately increased radiation sensitivity of SNB19 cells treated with both substances as compared to those treated with NVP-AUY922 alone (Figure 1D, Schedule II). The summarized data for both tested cell lines are shown in Figure 5. The large portions of cells in the S- and G_2_/M-phase in drug-free samples (Figure 5) indicate that the cell cultures were in the exponential growth phase at the beginning of the experiments. A 24-h incubation with PD184352 caused an enrichment of G_1_-phase cells from 40-50% to 60-70% in both cell lines. Upon 24-h incubation with NVP-AUY922, the fraction of cells in G2/M-phase increased to ~65%, whereas the S-phase fraction strongly decreased in both cell lines. After combined drug treatment for 24 h, the amount of cells in the S- and G_2_/M-phase was lower than after treatment with NVP-AUY922 alone, but still much higher than in the untreated control. Thirty min post-IR, the cell cycle distribution was almost identical to that in non-irradiated samples. In contrast, 24 hours post-IR, the drug-free samples of both cell lines, and especially SNB19 cells, irradiated with 8 Gy, showed a marked G_2_/M arrest. Combined treatment with the two inhibitors caused a weaker G_2_/M-arrest in irradiated cells than after treatment with NVP-AUY922 alone. Application of a single IR dose of 2 Gy did not cause any distortions in the cell cycle progression.

In case of treatment Schedule II (Figure 5C, 5D), 30 min after IR the cell-cycle distributions of drug-free and drug-treated cells were mostly the same, regardless of IR exposure. A G_2_/M arrest was observed 24 h after IR with 8 Gy in both cell lines but to a different extent (Figure 5C, 5D). Interestingly, although a 24-h incubation with PD184352 alone caused a strong G_1_ arrest, together with IR (8 Gy) under schedule II it led to a massive G_2_/M arrest in both tested cell lines, most notably in SNB19 cells (Figure 5D). As expected, NVP-AUY922 alone caused S-phase depletion and G_2_/M arrest irrespectively of IR exposure. In addition, after combined treatment with both substances the G_2_/M arrest in irradiated cells was comparable to that after treatment with NVP-AUY922 alone and IR.

To sum up, a 24-h incubation with PD184352 caused a G_1_ arrest, however, together with IR it caused a massive G_2_/M arrest and S-phase depletion under schedule II. Combined treatment with both drugs caused a strong G_2_/M arrest 24 h post-IR in tumor cells irradiated under both schedules, especially in the SNB19 cells treated under schedule II.

### Effects of inhibitors and radiation on late-stage apoptosis

To further explore the mechanisms underlying the radiation response of two tumor cell lines after MEK inhibition alone or in combination with Hsp90 inhibitor (Figure 1), we also analyzed the degree of late-stage apoptosis which was evaluated by the sub-G1 fraction. As seen in Supplementary Figure 6, the sub-G_1_ fraction was almost negligible at a time of IR but strongly increased in SNB19 cells treated under Schedule I with NVP-AUY922 alone or in combination with PD184352, most notably 24 h after IR (Supplementary Figure 6B). The same trend was observed in the respective samples of A549 cells, but, to a much lesser extent (Supplementary Figure 6A). The induction of late-stage apoptosis measured by the sub-G_1_ fraction was almost negligible in both cell lines treated with the inhibitors and/or irradiated under schedule II (Supplementary Figure 6C, 6D).

## DISCUSSION

Oncologists have combined chemotherapy and radiation treatment since the 1980s [27] and the combination of radiation and concurrent chemo- or molecularly targeted therapy has been convincingly shown to be superior to radiation alone in treatment of several cancer forms [28]. The efficacy of radiochemotherapy depends - among others factors - on the schedule of drug administration [28]. Particularly, the combination of gemcitabine followed by gefitinib (an inhibitor of the epidermal growth factor receptor) has been found to be more effective in tumor growth control than the reverse drug application [28].

The present study addresses two related questions: (*i*) whether the up-regulation of p-Erk and p-Akt proteins induced by MEK inhibition can be prevented by concomitant Hsp90 inhibition and (*ii*) whether the MEK inhibition can enhance the radiosensitizing effect of the Hsp90 inhibitor NVP-AUY922 in 2 tumor cell lines, *i.e*. lung carcinoma A549 and glioblastoma SNB19.

A major new finding of this study is that, depending on the drug-IR schedule, the MEK inhibitor PD184352 promoted either the radiosensitizing activity of NVP-AUY922 in the glioblastoma SNB19 cell line or a cytostatic effect in both tested cell lines. The effects were seen only if the MEK inhibitor was added to cells *shortly before IR* and cells were incubated with it up to 24 h thereafter (Schedule II), as evidenced by the colony counts shown in Figure 1. Interestingly, after treatment according to Schedule II neither inhibitor alone nor their combination exerted a radiosensitizing effect in A549 cells (Figure 1C). The lack of the radiosensitizing effect of NVP-AUY922 in A549 cells under this schedule, despite total depletion of p-MEK1/2, p-Erk1/2 and p-S6, is corroborated by the results of a previously published study [20]. Yet, as seen in the Supplementary Table 2, the PE values in the presence of either inhibitor decreased by 30-50% with respect to the drug-free control, which suggests that both drugs acted as cytostatics. In addition, comparison of further parameters, *i.e*. SF2 and D_10_ (Supplementary Table 2), revealed that under Schedule II PD184352 can significantly enhance the NVP-AUY922-mediated radiosensitization of the glioblastoma SNB19 cell line. At variance with Schedule II, a long-term pretreatment of cells with PD184352 (Schedule I) did not reveal this MEK inhibitor as a cytostatic agent nor was it found to increase the radiosensitizing effect of NVP-AUY922 (Figure 1).

**Figure 6:**
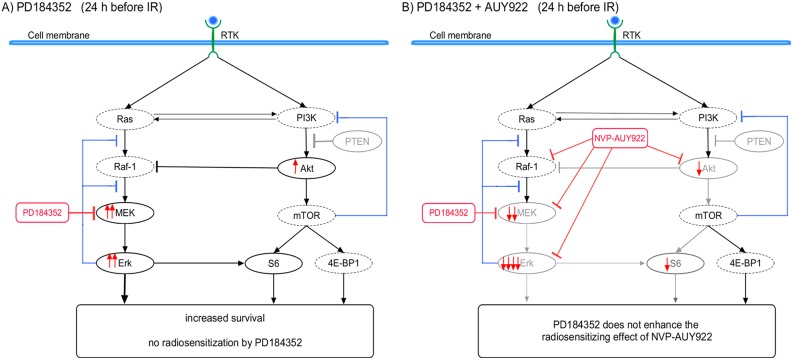

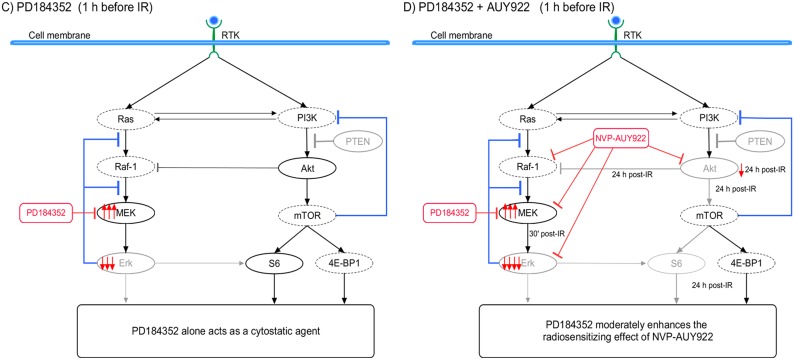
A simplified diagram of putative signaling pathways accountable for the differential responses of SNB19 tumor cells to MEK- and Hsp90-inhibition and IR used in two different drug-irradiation schedules Incubation of tumor cells with PD184352 for 24 h prior to IR **(A)** leads to a reactivation of the MAPK- and PI3K-pathways at the time of irradiation, most likely due to inhibition of the negative feedback loop mediated by ribosomal protein S6 (Figure 2). In contrast, after short time (1 h) incubation with PD184352, p-MEK is up-regulated whereas p-Erk is strongly depleted at the time of irradiation **(C)** as a result of the effective targeting of MEK by PD184352 (Figure 3). At the same time, p-Akt is not up-regulated. To summarize, long-term preincubation with PD184352 **(B)** caused no enhancement of the radiosensitizing effect of NVP-AUY922, whereas short-term preincubation **(D)** led to increased radiosensitization by a combination of the inhibitors compared to the Hsp90 inhibitor alone. (Size of protein names/symbols and line thickness indicate up- and down-regulation).

In order to elucidate the dependence of the radiosensitizing and/or cytostatical activity of PD184352 on the drug-IR schedule we thoroughly examined the expression of several key proteins of the PI3K pathway, along with the induction and repair of DNA damage, and the cell-cycle progression. The observed differences between the cellular responses to combined drug-IR treatment used under two drug-IR schedules can be explained by a simplified model illustrated in Figure 6. The model takes into account the different expressions of marker proteins of the MAPK and PI3K pathways (Figures 2, 3), which were dependent on the incubation time with the inhibitors before IR.

Surprisingly, we found that the long-term (24 h) incubation with PD184352 led to the *up-regulation* of p-Erk1/2 (Figure 2, left-hand side). The reactivation of p-Erk1/2 points toward the disruption of the negative feedback loops that normally down-regulates MAPK signaling, which in turn can paradoxically promote cell survival [29]. In addition, we observed the up-regulation of p-Akt in SNB19 cell line in the presence of PD184352 used under Schedule I. Activated Akt is widely recognized as the major mediator of cell survival, which inhibits apoptosis through several mechanisms [12], *e.g.* keeping mitochondrial integrity, phosphorylation and inactivation of proapoptotic BAD (Bcl-2-antagonist of cell death) and caspase 9 *etc*. [30]. BAD maintains Bcl-2 (B-cell lymphoma 2) and Bcl-xL function thereby inhibiting apoptosis mainly at the mitochondrial level by suppressing cytochrome *c* release [31]. The up-regulation of both PI3K- and MAPK-pathways at the time of IR would explain the lack of radiosensitization by PD184352 used under Schedule I (*i.e*. long-term pretreatment) and the absence of increased tumor cell killing in the presence of both substances (Figure 1A, 1B), as compared to the effects of NVP-AUY922 alone.

In contrast to Schedule I, the short-term (1 h) pretreatment with PD184352 (Schedule II) caused a *depletion* of the phosphorylated form of Erk1/2 (Figure 3). In addition, p-Akt was not up-regulated in samples treated with PD184352 alone or in combination with NVP-AUY922 (Figure 3). To sum up, the main advantage of Schedule II over Schedule I was in preventing the up-regulation of both p-Erk1/2 and p-Akt when PD184352 was given alone. Therefore, both proteins can be viewed as important markers of radiation sensitivity.

A further critical determinant of the radiation-induced cell death is the induction and repair of DNA DSBs, probed in this study by the expression of histone γH2AX (Figure 4). We found that the kinetics of DNA damage repair differed markedly between the two treatment protocols and the two tested cell lines. In cell samples pretreated with the PD184352 according to Schedule I, the initial DNA damage was very similar to that in drug-free samples, and the DNA damage completely recovered within 24 h post-IR. This finding can be explained by the lower radiosensitivity of the G_1_-arrested cells induced by prolonged treatment with the MEK-inhibitor alone. For the same reason, the DNA damage in the samples treated with both substances under Schedule I was much lower than that after treatment with NVP-AUY922 alone, which caused a stronger G_2_ arrest and subsequently more DNA damage than combined treatment with two inhibitors. In contrast, cells treated with both substances according to Schedule II showed high residual DNA damage levels up to 24 h after IR, which were comparable to those in cells treated with the Hsp90 inhibitor alone, at least in SNB19 cells. At the time of IR under schedule II the MEK inhibitor did not induce G_1_ arrest yet. However, the DNA damage in A549 cells treated with the inhibitors under schedule II, either alone or in combination almost recovered to control levels.

In addition to the above mentioned reasons, the differences between the two schedules in the radiosensitivity of the drug-treated tumor cells (Figure 1) can partly be explained by peculiarities of the cell cycle phase distribution. Thus, the long-term treatment with PD184352 (Schedule I) prior to IR leads cells to reside predominantly in the G_1_ phase (Figure 5), which is known to be the most radioresistant phase of the cell cycle. Interestingly, combined PD184352-IR treatment under Schedule II caused a strong G_2_/M block 24 h after IR. Because the two tested tumor cell lines are different in their mutational status of *PTEN* and *p53* (*i.e*. mut *PTEN* and mut *p53* in SNB19 *vs*. wt *PTEN* and wt *p53* in A549), we cannot definitely conclude whether the radiosensitizing effect of PD184352 in combination with NVP-AUY922 was associated with either *PTEN* or *p53* mutations. In addition, the tested cells lines also differ in their *kRas* mutational status (mut *kRas* in A549 *vs*. wt *kRas* SNB19). Therefore, the relationship between the radiosensitivity of tumor cells to the inhibitors and their *PTEN*, *p53* and *kRas* status needs further investigation.

To sum up, our data demonstrate an enhanced radiosensitivity in tumor cells pretreated with MEK and Hsp90 inhibitors shortly before IR. The findings corroborate the importance of the drug administration schedule for radiosensitization of tumor cells reported previously [28, 32]. The complex mechanisms underlying the increased radiosensitization by PD184352 and NVP-AUY922 inhibitors apparently involve multiple, cell line-specific pathways that lead to the down-regulation of the MAPK-pathway and prevent the up-regulation of the PI3K-pathway at the moment of IR, followed by a strong G_2_/M arrest and protracted DNA damage repair 24 h thereafter. In contrast, long-term treatment with PD184352 before IR failed to enhance the radiosensitizing effect of the Hsp90 inhibitor. Possible reasons for the failure can be the drug-mediated activation of the prosurvival MAPK- and PI3K-pathways, G_1_ arrest during IR exposure, and almost unimpaired DNA damage repair. Yet the observed strong arrest of tumor cells in G_1_ phase justifies the use of MEK inhibitors as potential cytostatic drugs, and particularly multiple MEK inhibitors are currently being tested in clinical trials Phase I-II (https://www.clinicaltrials.gov). Finally, our *in vitro* data reveal the importance of the duration of MEK inhibition before IR for the radiosensitization of tumor cells and underline the fact that the therapeutic window for treatment with MEK inhibitors needs to be carefully defined, or a combination of inhibitors should be considered.

## MATERIALS AND METHODS

### Cells

The lung carcinoma A549 and glioblastoma SNB19 cell lines were obtained from the ATCC (Manassas, VA) and cultured under standard conditions (5% CO_2_, 37°C) in complete growth medium (CGM) containing DMEM supplemented with 10% fetal bovine serum. The A549 cell line bears mutation in *kRas*, *SMARCA4*, *STK12*, whereas SNB19 is mutated for *p53* and *PTEN*, and both cell lines are mutated for *CDKN2A*, *CDKN2a(p14)* [http://www.sanger.ac.uk/genetics/CGP/cosmic/, COSMIC, Catalogue of Somatic Mutations In Cancer].

### Drug treatment

Both drugs, PD184352 and NVP-AUY922, were obtained from Selleckchem (Absource Diagnostics GmbH, Munich, Germany). The drugs were freshly diluted from frozen aliquots in DMSO stored at -20°C. Cells were treated with either PD184352 (2 μM, [33]), NVP-AUY922 (50 nM, [34]), or both substances, according to two different time schedules (Supplementary Figure 1). In Schedule I the substances were added 24 h before IR and washed out shortly before IR. Under Schedule II the drugs were added 1 h prior to IR and remained in CGM up to 24 h post-IR. Cells treated in parallel with respective amounts of DMSO served as controls.

### X-ray irradiation

Irradiation was performed at room temperature using a 6 MV Siemens linear accelerator (Siemens, Concord, CA, USA) at a dose rate of 2 Gy/min. After irradiation, cells were kept in CGM for the indicated time until harvest.

### Colony survival assay

Cell survival was assessed by colony formation as previously described [35]. Subconfluent monolayers of control and inhibitor-treated cells were irradiated in culture flasks filled with CGM at room temperature by graded single doses (0 - 8 Gy), seeded either immediately or 24 h post-IR in Petri dishes and then cultured for 10-12 days in CGM. Four replicates were performed for each radiation dose, and the experiments were repeated at least four times. After 2 weeks, the cells were fixed and stained with crystal violet (0.6%). Macroscopic colonies containing at least 50 cells were scored as survivors. The mean clonogenic survival data for each cell line were fitted to the LQ model (Equation 1):

$$SF=\exp\left(-aX-\beta X^{2}\right)$$ (Equation 1)

where, *SF* is the survival fraction, *X* is the irradiation dose, α and β are the fitted parameters.

### Western blotting

For immunoblot assays, whole-cell lysates were prepared either 30 min or 24 h post-IR, according to standard procedures. Samples equivalent to 20 - 40 μg of protein were separated using 4-12% SDS-polyacrylamide pre-cast gels (Invitrogen, Karlsruhe, Germany) and transferred to nitrocellulose membranes. For protein detection, membranes were incubated with respective primary and species-specific peroxidase-labeled secondary antibodies according to standard protocols. The levels of protein expression were quantified using the software ImageJ (NIH, Bethesda, MD, USA) and normalized to β-actin levels.

### Antibodies

The primary and secondary antibodies are specified in Supplementary Information.

### DNA damage and cell-cycle measurements by flow cytometry

Non-treated and drug-treated cell cultures were irradiated as subconfluent monolayers in CGM at room temperature. The cells were then incubated under standard conditions and analyzed by flow cytometry 30 min and 24 h after IR exposure. For analysis, cells were trypsinized, washed twice in PBS, fixed and stained for γH2AX according to a protocol described elsewhere [36]. The cells were then counterstained with propidium iodide (PI, Sigma P-4170, 10 μg/ml) in the presence of ribonuclease A (Sigma R-5250, 25 μg/ml) as described elsewhere [37]. At least 20,000 cells were assayed for either histone γH2AX or DNA distribution using a flow cytometer FACSCantoII (Becton Dickinson, San Jose, CA, USA). Cellular green (histone γH2AX) or red (DNA-PI) fluorescence was acquired in logarithmic or linear mode, respectively. The output data were presented as one-dimensional histograms, *i.e*. the distributions of histone γH2AX or PI-DNA signals within cell samples, and were analyzed using the Flowing Software program obtained from P. Terho (Turku Centre for Biotechnology, Turku, Finland) and the software ModFit LT (Verity Software House, Topsham, ME). In addition, the sub-G_1_ fraction was evaluated to assess the late-stage apoptosis.

### Statistics

Data are presented as means (± SD or ± SE). Mean values were compared by the Student's *t*-test. The threshold of statistical significance was set at *P* < 0.05. Statistics and fitting of experimental data were performed with Origin 8.5 (Microcal, Northampton, MA, USA).

## SUPPLEMENTARY MATERIALS FIGURES AND TABLES



## Abbreviations

BAD: Bcl-2-antagonist of cell death
Bcl: B-cell lymphoma
CGM: complete growth medium
D_10_: radiation dose required to reduce clonogenic survival by 10%
DSB: double-strand breaks
Erk: extracellular signal-regulated kinase
Hsp90: heat shock protein 90
IR: ionizing radiation
LHS: left-hand side
LQ: linear-quadratic
MAPK: mitogen-activated protein kinase (MAPK) kinase (MEK)
MEK: mitogen-activated protein kinase (MAPK) kinase
PE: plating efficiency
PI: propidium iodide
PI3K: phosphoinositid-3-kinase
RAF: Rat fibrosarcoma protein
RAS: Rat sarcoma protein
RHS: right-hand side
SF2: surviving fraction at 2 Gy.
