# Supplementary material for: MEK-inhibitor PD184352 enhances the radiosensitizing effect of the Hsp90 inhibitor NVP-AUY922: the role of cell type and drug-irradiation schedule
Source: Oncotarget. 2018 Dec 21;9(100):37379–92. doi: 10.18632/oncotarget.26436 (PMC6324777; doi:10.18632/oncotarget.26436)
Supplement: Supplementary file 1 [file oncotarget-09-37379-s001.pdf]

MEK-inhibitor PD184352 enhances the radiosensitizing effect of the Hsp90 inhibitor NVP-AUY922: the role of cell type and drug-irradiation schedule

SUPPLEMENTARY MATERIALS

Antibodies

The primary antibodies used were: rabbit polyclonal anti-Akt, mouse monoclonal anti-phospho-Akt (Ser473), mouse monoclonal anti-S6 ribosomal protein (54D2), rabbit polyclonal anti-phospho-S6 (Ser240/244), rabbit monoclonal anti-MEK1/2 (47E6), rabbit monoclonal anti-phospho-MEK1/2 (Ser217/221), rabbit polyclonal anti-p44/42MAPK (Erk1/2), rabbit monoclonal anti-phospho-p44/42 MAPK (Erk1/2) (Thr202/Tyr204) (all from Cell Signaling Technologies Inc., Danvers, MA, USA), mouse monoclonal anti-Hsp70, mouse monoclonal anti-Hsp90 (BD, Heidelberg, Germany) mouse monoclonal anti-β-actin (Sigma, Deisenhofen, Germany) and mouse monoclonal anti-phospho-histone H2AX (Ser139) FITC-conjugate (Millipore, Schwalbach, Germany). Secondary species-specific antibodies for western blot were labelled with horseradish-peroxidase (DAKO, Hamburg, Germany).

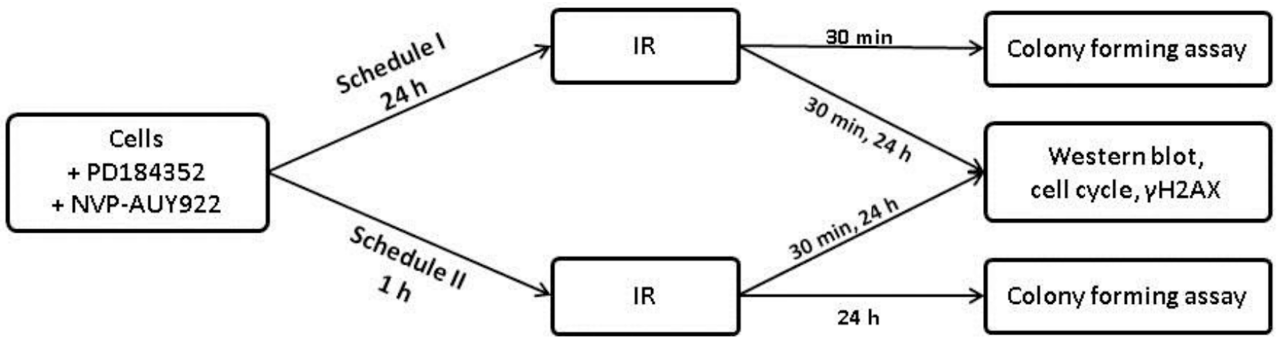

Supplementary Figure 1: Two different drug-irradiation (IR) schedules were used in the study. PD184352 and NVP-AUY922 were added to the tumor cell cultures either for 24 h (Schedule I) and washed out shortly before IR. Schedule II: PD184352 and NVP-AUY922 were added to the cell cultures 1 h before IR (2 and 8 Gy) and kept in culture medium up to 24 h post-IR. Cells treated in different schedules were then analyzed at indicated times by colony-forming ability, expression of marker proteins, DNA damage and cell-cycle progression.

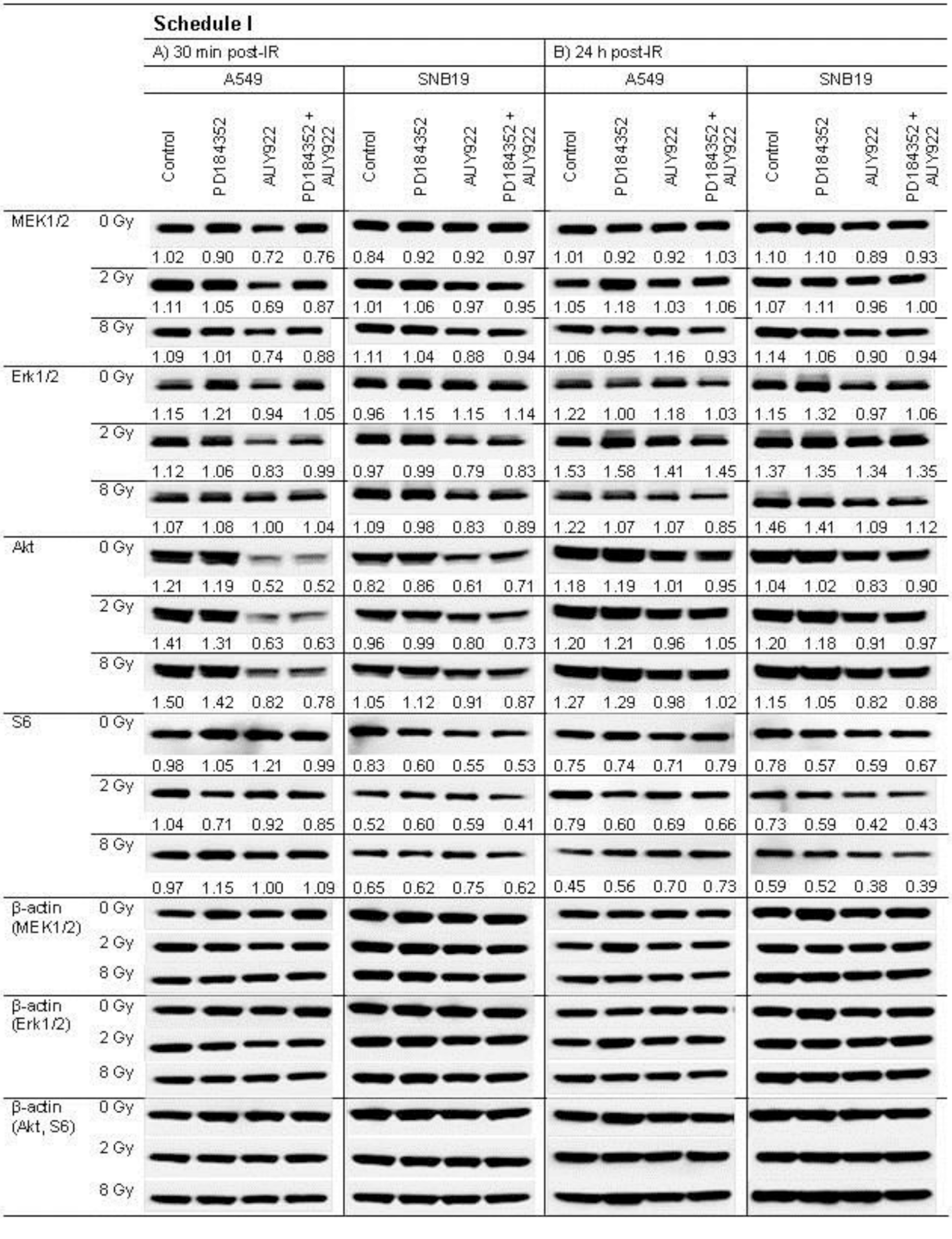

Supplementary Figure 2: Effects of PD184352, NVP-AUY922 and IR on the expression levels of marker proteins in A549 and SNB19 cell lines detected 30 min and 24 h post-IR. Cells were treated with PD184352, NVP-AUY922 and IR in schedule I. For details, *see* legend to Figure 2.

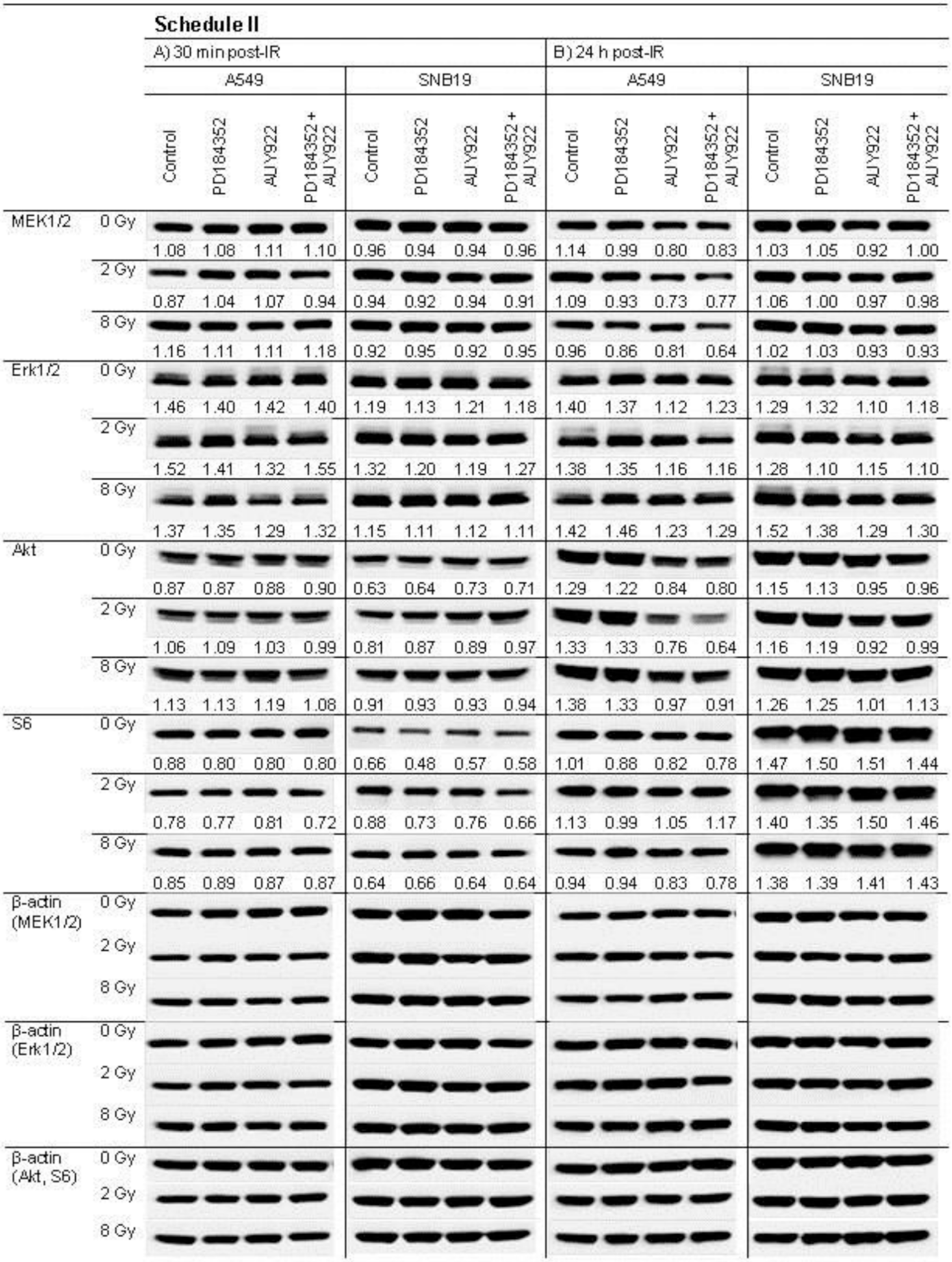

Supplementary Figure 3: Effects of PD184352, NVP-AUY922 and IR on the expression levels of proteins in A549 and SNB19 cell lines detected 30 min and 24 h post-IR. Cells were treated with PD184352, NVP-AUY922 and IR in schedule II. For details, *see* legend to Figure 3.

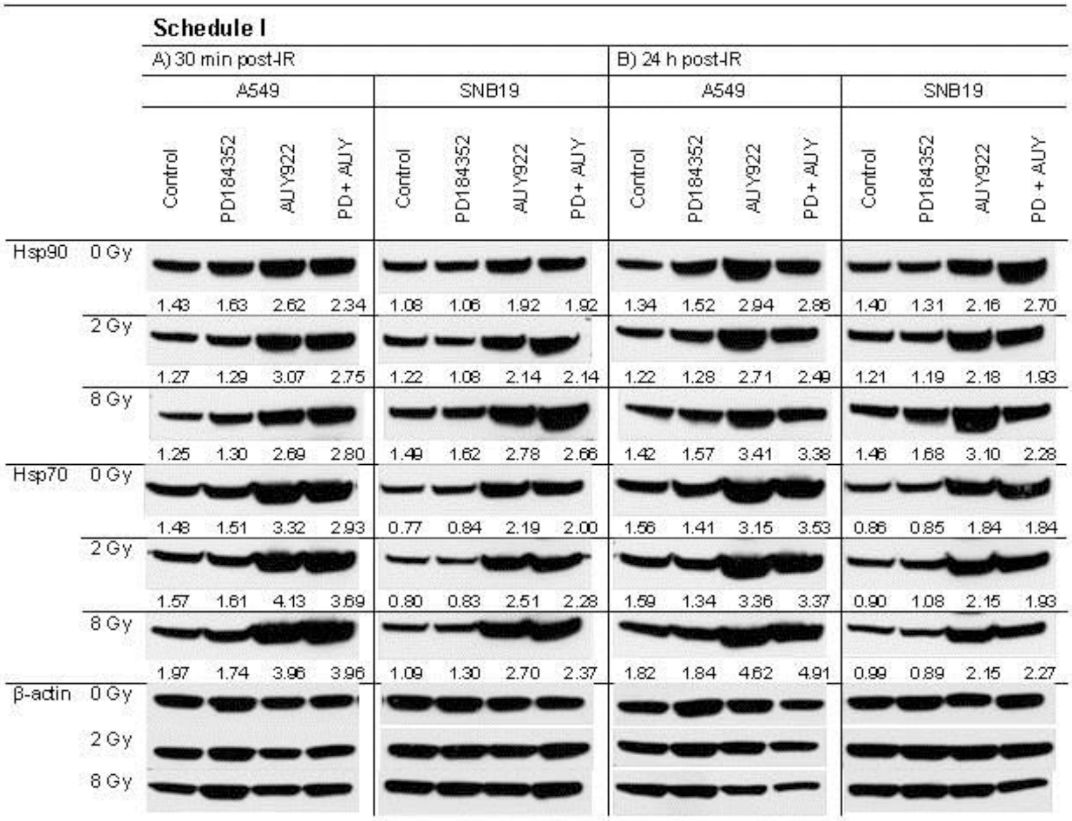

Supplementary Figure 4: Effects of PD184352, NVP-AUY922 and IR on the expression levels of Hsp90 and Hsp70 proteins in A549 and SNB19 cell lines detected 30 min and 24 h post-IR. Cells were treated with PD184352, NVP-AUY922 and IR in schedule I. For details, see legend to Figure 2.

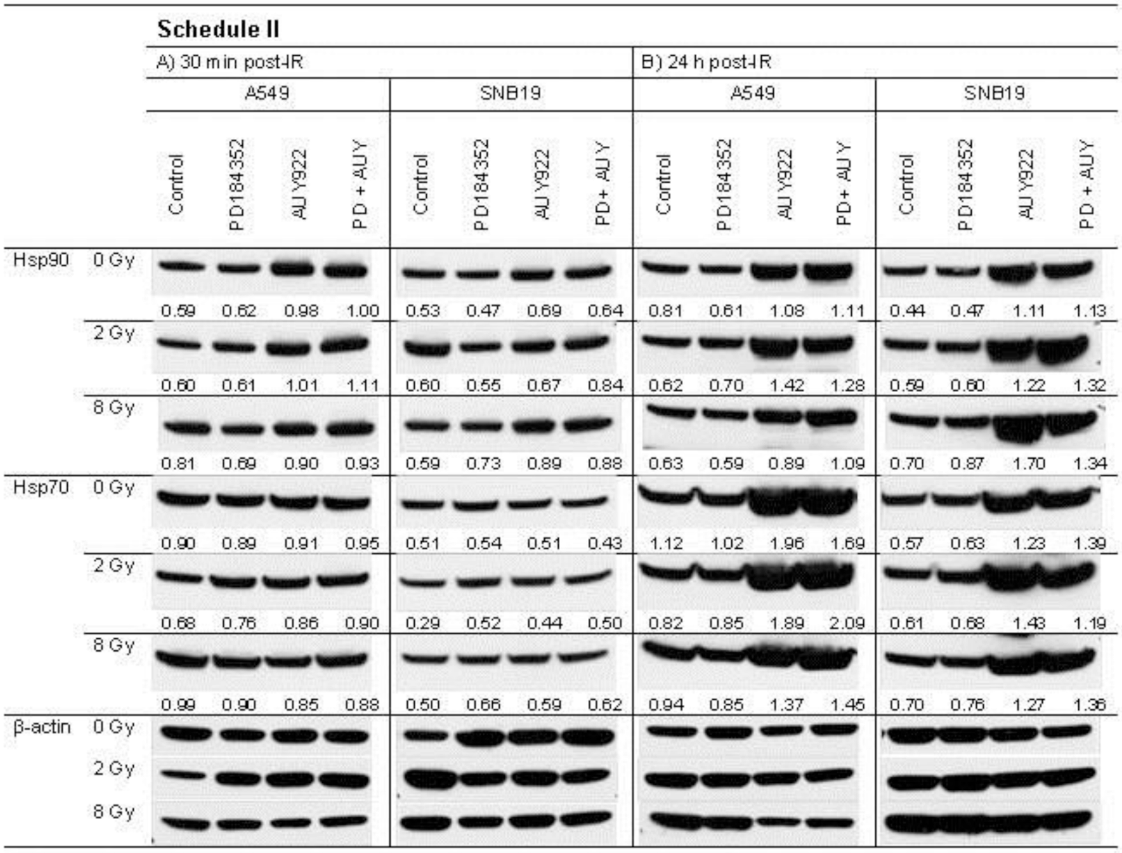

Supplementary Figure 5: Effects of PD184352, NVP-AUY922 and IR on the expression levels of Hsp90 and Hsp70 proteins in A549 and SNB19 cell lines detected 30 min and 24 h post-IR. Cells were treated with PD184352, NVP-AUY922 and IR in schedule II. For details, see legend to Figure 3.

Supplementary Table 1: Cloning efficiencies and radiosensitivity parameters[a] of *in vitro* irradiated tumor cell lines untreated and pretreated with the PD-184352 and NVP-AUY922 for 24 h before IR and replated immediately after IR according to Schedule I

Cell line	Plating efficiency	SF2	D_{10} (Gy)[b]	IF_{10}[c] (D_{10} control)/ (D_{10}+inh.)
**A549 – contr.**	0.73±0.03	0.72±0.06	6.5±0.4	1.0
+ PD184352	0.66±0.04	0.70±0.05	6.7±0.5	1.0±0.1
+ AUY922	0.42±0.04	0.34±0.06	3.8±0.3	1.8±0.2
+ both drugs	0.42±0.05	0.35±0.04	3.8±0.2	1.8±0.2
**SNB19 – contr.**	0.20±0.02	0.66±0.02	6.7±0.5	1.0
+ PD184352	0.18±0.03	0.71±0.03	5.9±0.2	1.1±0.1
+ AUY922	0.13±0.03	0.31±0.06	3.3±0.4	2.1±0.1
+ both drugs	0.11±0.03	0.29±0.05	3.2±0.4	2.1±0.2

[a]Mean (± SE) from at least five independent experiments;
[b]D_{10} is the radiation dose required to reduce clonogenic survival by 10%;
[c]The growth inhibition factor IF_{10} was calculated as (D_{10} control)/(D_{10}+inh.)
For detailed description, *see* legend to Figure 1.

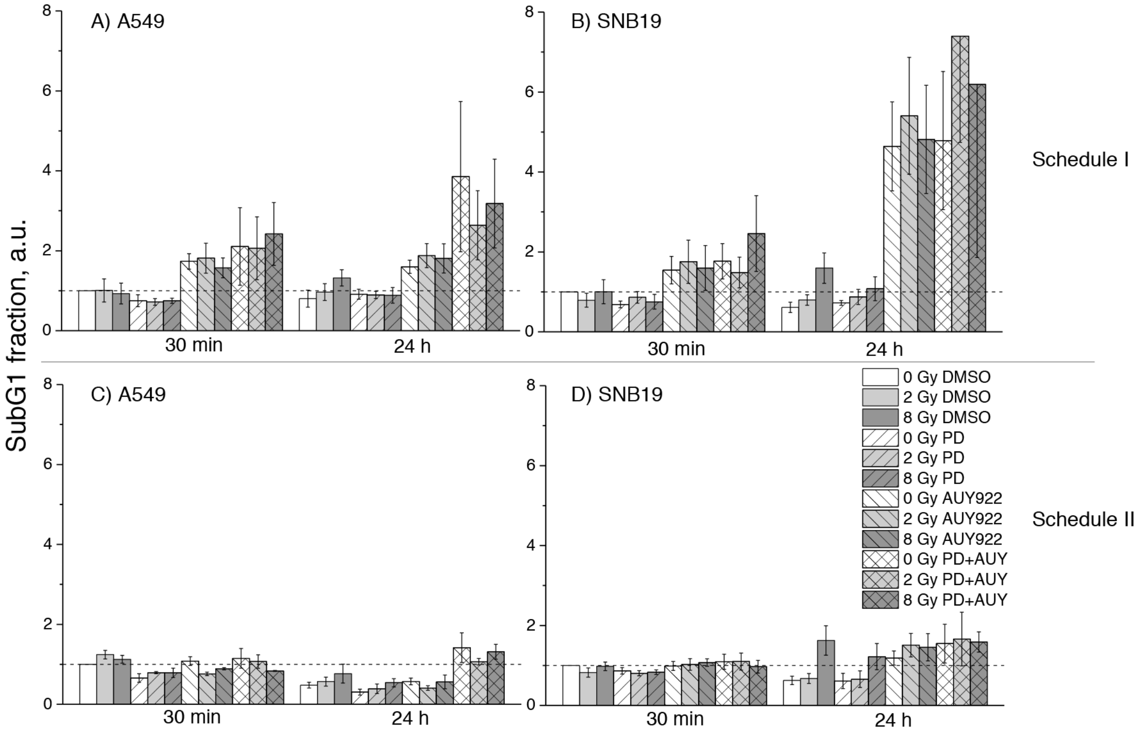

Supplementary Figure 6: Simultaneous inhibitors and radiation treatment caused late-stage apoptosis measured by sub-G_1 fraction. (A-D) Flow-cytometric analyses of the DNA content distribution in A549 and SNB19 cells subjected to drug-IR treatments were performed 30 min or 24 h after IR. The cells were detached with trypsin, treated with saponin and RNAse, stained with propidium iodide and then analyzed for red fluorescence by flow cytometry. The samples include both floating and trypsinized cells. Changes in the sub-G_1 fraction are depicted as folds of increase (means ± SE, n=3), normalized to respective non-irradiated drug-free controls (30 min post-IR). The sub-G1 fraction includes hypodiploid nuclei and debris, computed from the flow cytograms by means of the Flowing Software for n=3 independent experiments. For details, *see* legend to Figure 5.

Supplementary Table 2: Cloning efficiencies and radiosensitivity parameters[a] of *in vitro* irradiated tumor cell lines untreated and pretreated with the PD-184352 and NVP-AUY922 for 1 h before IR and replated 24 h post-IR according to Schedule II

**Cell line**	**Plating efficiency**	**SF2**	**D_{10} (Gy)[b]**	**IF_{10}[c] (D_{10} control)/ (D_{10}+inh.)**
**A549 – contr.**	0.83±0.06	0.72±0.06	7.6±0.6	1.0
+ PD184352	0.62±0.08	0.71±0.07	8.5±0.9	0.9±0.1
+ AUY922	0.49±0.09	0.60±0.06	7.8±0.6	1.0±0.1
+ both drugs	0.39±0.11	0.58±0.05	7.3±0.3	1.0±0.1
**SNB19 – contr.**	0.28±0.10	0.74±0.03	7.8±0.5	1.0
+ PD184352	0.16±0.04	0.77±0.03	7.7±0.6	1.0±0.1
+ AUY922	0.10±0.03	0.40±0.05	4.3±0.4	1.8±0.3
+ both drugs	0.15±0.06	0.33±0.02	3.6±0.2	2.2±0.3

For details, *see* Supplementary Table 1.

Supplementary Table 3: Cell cycle-phase distribution in A549 tumor cells treated with PD184352 (PD), NVP-AUY922 (AUY) and IR according to Schedule I. Thirty minutes and 24 h after IR cells were fixed, permeabilized, stained with PI, and analyzed for DNA content by flow cytometry

Cell line	Treatment modality			G0/G1 (%)	S (%)	G2/M (%)	G2/G1
**A549**	30 min	0 Gy	Control	51.1±3.1	34.3±3.6	14.7±1.3	0.3
			PD	63.5±2.9	25.7±2.2	10.8±1.1	0.2
			AUY	31.9±7.1	4.8±0.8	63.3±7.6	2.0
			PD+AUY	41.2±7.5	2.1±0.5	56.7±7.9	1.4
		2 Gy	Control	52.7±1.7	34.6±1.9	12.7±0.7	0.2
			PD	64.3±3.4	25.4±1.9	10.3±1.5	0.2
			AUY	31.4±5.7	3.4±1.3	65.2±4.4	2.1
			PD+AUY	39.7±5.0	4.1±1.9	57.2±4.9	1.5
		8 Gy	Control	53.6±2.0	33.7±2.0	12.7±0.4	0.2
			PD	65.1±3.4	24.7±2.0	10.2±1.5	0.2
			AUY	29.8±6.1	2.8±0.8	67.4±5.3	2.3
			PD+AUY	38.6±6.9	2.2±0.4	59.3±7.3	1.5
	24 h	0 Gy	Control	49.2±0.7	36.7±0.5	14.2±0.2	0.3
			PD	53.7±2.6	32.0±3.1	14.3±0.5	0.3
			AUY	45.6±4.5	23.8±0.8	30.6±3.9	0.7
			PD+AUY	36.8±13.8	31.4±6.9	31.8±7.1	0.9
		2 Gy	Control	51.6±1.2	33.7±1.5	15.0±0.9	0.3
			PD	59.6±1.2	27.0±0.6	13.4±0.8	0.2
			AUY	32.2±4.8	20.8±7.2	47.3±5.2	1.5
			PD+AUY	29.4±4.5	24.2±7.0	46.5±2.7	1.6
		8 Gy	Control	55.0±3.7	12.2±3.9	32.9±5.4	0.6
			PD	64.2±4.2	11.7±0.9	24.2±4.0	0.4
			AUY	25.4±1.8	13.8±6.8	60.7±7.4	2.4
			PD+AUY	32.6±2.3	15.5±6.1	51.9±8.3	1.6

Data are presented as means (± SD) from at least three independent experiments. For detailed description, *see* legend to Figure 5.

Supplementary Table 4: Cell cycle-phase distribution in SNB19 tumor cells treated with PD184352 (PD), NVP-AUY922 (AUY) and IR according to Schedule I. Thirty minutes and 24 h after IR cells were fixed, permeabilized, stained with PI, and analyzed for DNA content by flow cytometry

Cell line	Treatment modality			G0/G1 (%)	S (%)	G2/M (%)	G2/G1
**SNB19**	30 min	0 Gy	Control	45.4±1.7	33.0±0.5	21.6±2.1	0.5
			PD	59.7±3.4	18.9±1.4	21.4±2.1	0.4
			AUY	27.7±2.1	14.8±3.1	57.6±1.4	2.1
			PD+AUY	31.2±1.5	11.2±2.0	57.6±0.8	1.8
		2 Gy	Control	46.0±1.1	33.7±0.5	20.3±1.4	0.4
			PD	56.5±6.4	26.5±6.3	17.1±2.1	0.3
			AUY	27.0±1.8	16.0±3.3	57.0±2.3	2.1
			PD+AUY	31.3±1.4	12.6±3.6	56.1±3.1	1.8
		8 Gy	Control	46.7±0.9	33.3±1.2	20.0±2.0	0.4
			PD	60.7±4.2	19.6±2.4	19.7±1.9	0.3
			AUY	27.6±2.3	15.5±3.5	57.0±1.7	2.1
			PD+AUY	34.0±2.3	12.9±2.6	53.2±1.6	1.6
	24 h	0 Gy	Control	46.1±0.9	31.3±0.8	22.6±0.9	0.5
			PD	48.9±4.9	24.2±4.1	26.9±1.0	0.5
			AUY	10.0±2.7	33.9±5.7	56.1±8.3	5.6
			PD+AUY	13.8±3.4	41.6±3.3	44.7±6.4	3.2
		2 Gy	Control	46.5±0.7	32.1±0.5	21.5±0.4	0.5
			PD	47.1±4.3	25.0±3.2	27.8±1.8	0.6
			AUY	4.6±1.4	30.3±6.4	65.1±7.8	14.2
			PD+AUY	8.5±3.6	36.8±5.0	54.6±8.0	6.4
		8 Gy	Control	48.4±4.5	9.3±1.5	42.3±5.5	0.9
			PD	42.3±6.4	11.7±2.4	46.0±4.5	1.1
			AUY	1.0±0.6	26.4±4.5	72.6±4.5	72.6
			PD+AUY	1.2±0.4	37.4±6.8	61.4±6.8	51.2

Data are presented as means (± SD) from at least three independent experiments. For detailed description, *see* legend to Figure 5.

Supplementary Table 5: Cell cycle-phase distribution in A549 tumor cells treated with PD184352 (PD), NVP-AUY922 (AUY) and IR according to Schedule II. Thirty minutes and 24 h after IR cells were fixed, permeabilized, stained with PI, and analyzed for DNA content by flow cytometry

Cell line	Treatment modality			G0/G1 (%)	S (%)	G2/M (%)	G2/G1
**A549**	30 min	0 Gy	Control	46.6±1.3	36.7±1.4	16.7±0,0	0.4
			PD	47.3±2.5	36.8±2.5	16.0±0.4	0.3
			AUY	48.0±2.7	35.9±1.9	15.3±1.1	0.3
			PD+AUY	47.8±2.9	36.9±1.5	15.3±2.0	0.3
		2 Gy	Control	46.9±2.9	36.5±1.8	16.5±1.7	0.4
			PD	49.0±2.3	34.5±1.4	16.5±0.9	0.3
			AUY	49.1±2.4	34.5±2.0	16.4±0.7	0.3
			PD+AUY	49.4±2.1	35.6±1.6	15.1±1.2	0.3
		8 Gy	Control	49.1±2.4	35.3±1.1	15.5±1.4	0.3
			PD	48.1±1.9	36.5±1.2	15.4±0.7	0.3
			AUY	48.6±3.0	36.8±1.6	14.6±1.7	0.3
			PD+AUY	48.7±2.5	36.3±2.0	15.0±1.1	0.3
	24 h	0 Gy	Control	52.1±2.3	34.5±2.2	13.4±1.2	0.3
			PD	66.3±1.6	21.7±1.9	12.0±1.5	0.2
			AUY	34.4±3.2	2.4±0.5	63.5±3.6	1.9
			PD+AUY	43.2±5.5	2.2±0.4	54.6±5.7	1.3
		2 Gy	Control	50.7±2.1	32.9±3.7	16.3±1.6	0.3
			PD	74.7±3.9	13.0±1.2	12.4±2.9	0.2
			AUY	38.9±1.1	1.2±0.1	59.9±1.1	1.5
			PD+AUY	40.6±2.1	2.2±0.5	57.1±1.6	1.4
		8 Gy	Control	57.7±3.3	11.8±2.5	30.4±5.6	0.5
			PD	60.3±4.7	5.4±0.8	34.4±5.2	0.6
			AUY	36.6±1.1	1.5±0.3	61.9±0.8	1.7
			PD+AUY	39.1±2.9	2.6±0.5	58.3±2.5	1.5

Data are presented as means (± SD) from at least three independent experiments. For detailed description, *see* legend to Figure 5.

Supplementary Table 6: Cell cycle-phase distribution in SNB19 tumor cells treated with PD184352 (PD), NVP-AUY922 (AUY) and IR according to Schedule II. Thirty minutes and 24 h after IR cells were fixed, permeabilized, stained with PI, and analyzed for DNA content by flow cytometry

Cell line	Treatment modality			G0/G1 (%)	S (%)	G2/M (%)	G2/G1
**SNB19**	30 min	0 Gy	Control	26.6±1.3	49.5±4.5	23.9±5.2	0.9
			PD	26.6±1.7	50.9±3.1	22.5±4.0	0.8
			AUY	27.0±1.9	50.3±3.8	21.9±5.0	0.8
			PD+AUY	27.1±2.7	51.0±4.6	21.9±5.1	0.8
		2 Gy	Control	27.0±1.4	50.6±5.0	22.4±5.4	0.8
			PD	27.1±2.4	51.0±4.6	21.9±5.8	0.8
			AUY	27.7±2.6	50.4±4.7	21.8±4.9	0.8
			PD+AUY	27.0±2.6	51.3±5.7	21.7±5.2	0.8
		8 Gy	Control	27.8±1.7	49.9±4.9	22.3±4.7	0.8
			PD	27.4±2.6	51.8±4.0	20.8±5.0	0.8
			AUY	26.9±2.6	51.6±4.6	21.5±5.4	0.8
			PD+AUY	26.9±2.8	51.3±4.4	21.7±4.1	0.8
	24 h	0 Gy	Control	43.3±1.7	33.9±3.3	22.8±1.6	0.5
			PD	59.4±1.6	18.2±1.9	22.4±1.1	0.4
			AUY	27.5±3.3	12.8±2.1	59.8±1.3	2.2
			PD+AUY	32.1±3.8	8.9±1.7	59.0±2.2	1.8
		2 Gy	Control	40.7±2.6	39.5±0.2	19.8±2.7	0.5
			PD	58.5±1.2	22.3±2.0	19.2±2.4	0.3
			AUY	15.0±2.0	8.7±1.5	76.3±1.1	5.1
			PD+AUY	16.4±3.3	16.0±9.5	67.6±6.6	4.1
		8 Gy	Control	35.8±3.2	11.3±2.4	52.8±5.3	1.5
			PD	44.8±3.1	7.2±1.4	48.0±4.2	1.1
			AUY	6.4±1.4	9.5±1.4	84.1±1.4	13.1
			PD+AUY	8.5±2.6	7.8±1.4	83.7±2.5	9.8

Data are presented as means (± SD) from at least three independent experiments. For detailed description, *see* legend to Figure 5.
